# Decrease in serum procalcitonin levels over time during treatment of acute bacterial meningitis

**DOI:** 10.1186/cc3722

**Published:** 2005-05-20

**Authors:** Alain Viallon, Pantéa Guyomarc'h, Stéphane Guyomarc'h, Bernard Tardy, Florianne Robert, Olivier Marjollet, Anne Caricajo, Claude Lambert, Fabrice Zéni, Jean-Claude Bertrand

**Affiliations:** 1 Emergency and Intensive Care Units, Bellevue Hospital, Saint-Etienne, France; 2 Microbiology Laboratory, Bellevue Hospital, Saint-Etienne, France; 3 Immunology Laboratory, Bellevue Hospital, Saint-Etienne, France

## Abstract

**Introduction:**

The aim of this study was to describe the change in serum procalcitonin levels during treatment for community-acquired acute bacterial meningitis.

**Methods:**

Out of 50 consecutive patients presenting with bacterial meningitis and infection at no other site, and who had received no prior antibiotic treatment, 48 had a serum procalcitonin level above 0.5 ng/ml on admission and were enrolled in the study.

**Results:**

The mean age of the patients was 55 years, and mean Glasgow Coma Scale score on admission was 13. The time from symptom onset to admission was less than 24 hours in 40% of the patients, 24–48 hours in 20%, and more than 48 hours in 40%. The median (interquartile) interval between admission and initial antibiotic treatment was 160 min (60–280 min). Bacterial infection was documented in 45 patients. Causative agents included *Streptococcus pneumoniae *(*n *= 21), *Neisseria meningitidis *(*n *= 9), *Listeria monocytogenes *(*n *= 6), other streptococci (*n *= 5), *Haemophilus influenzae *(*n *= 2) and other bacteria (*n *= 2). The initial antibiotic treatment was effective in all patients. A lumbar puncture performed 48–72 hours after admission in 34 patients showed sterilization of cerebrospinal fluid. Median (interquartile) serum procalcitonin levels on admission and at day 2 were 4.5 (2.8–10.8) mg/ml and 2 (0.9–5.0) mg/ml, respectively (*P *< 0.0001). The corresponding values for C-reactive protein were 120 (21–241) mg/ml and 156 (121–240) mg/ml, respectively. Five patients (10%) died from noninfectious causes during their hospitalization.

**Conclusions:**

Serum procalcitonin levels decrease rapidly with appropriate antibiotic treatment, diminishing the value of lumbar puncture performed 48–72 hours after admission to assess treatment efficacy.

## Introduction

Community-acquired acute bacterial meningitis (ABM) in adults remains a serious disease, with mortality rates of 10–25% [1,2]. In the context of emergency presentation, the management decisions to be made once the diagnosis has been established concern the initial antibiotic treatment [2], adjuvant therapies [3,4] and treatment of organ failure [5].

Antibiotic treatment must be started rapidly [6] and must be appropriate, particularly when risk factors are present [7,8], although the timing of antibiotic therapy initiation does not appear to be an independent prognostic factor [9-11]. The choice of antibiotic treatment, addressed in numerous national [12] and international [2,13-15] recommendations, is based on aetiological indices, risk factors, the results of direct examinations and knowledge of bacterial ecology.

The efficacy of this initial antibiotic therapy is assessed on the basis of clinical course of the disease and analysis of cerebrospinal fluid (CSF) samples obtained 48–72 hours after the start of treatment, when available, although cytochemical CSF parameters appear to be little modified by appropriate antibiotic treatment [16]. A marker that can demonstrate efficacy at an earlier stage would be extremely useful.

In 1993 Assicot and coworkers [17] demonstrated the value of serum procalcitonin (PCT) as a marker of infectious states of bacterial orgin in neonates and infants, as well as the rapid decrease in its concentration with appropriate antibiotic treatment. The aim of the present study was to describe the variation in serum PCT levels over time during the treatment of ABM.

## Materials and methods

### Patients

This was a prospective study and included patients admitted to the adult emergency department with community-acquired bacterial meningitis between January 1997 and October 2003. The demographic and clinical characteristics of the patients were recorded on admission.

Bacterial meningitis was diagnosed if pathogenic bacteria were detected in the CSF. In the absence of documented evidence of bacterial infection, this diagnosis was made if the polymorphonuclear leucocyte count in the CSF exceeded 250/mm^3 ^and the CSF/serum glucose ratio was below 0.4, with a compatible clinical state, necessitating antibiotic treatment for 7 days or longer. Patients presenting with a further site of infection in addition to meningitis on admission, having received prior antibiotic treatment for more than 2 consecutive days or showing a serum PCT level of 0.5 mg/ml or less, were excluded from the study.

### Laboratory tests

Blood samples for C-reactive protein (CRP), PCT, fibrinogen, lactate and creatinine assays, and complete blood count were taken on admission, then once daily during the first week. Lumbar puncture (for total and polymorphonuclear leucocyte count and assay of proteins, lactate and glucose) and bacteriological sampling (blood cultures) were performed before starting the initial antibiotic treatment. These tests could be repeated between 48 and 72 hours later at the discretion of the clinician.

The interval between admission and administration of the first dose of antibiotic was recorded. Bacterial sensitivity to antibiotics was routinely tested by determining the minimum inhibitory concentrations (MICs) of penicillin, amoxicillin, cefotaxime and ceftriaxone. With regard to penicillin, bacteria were considered to be sensitive if the MIC was 0.1 mg/l or less, of intermediate resistance if the MIC was above 0.1 mg/l but no greater than 1 mg/l, and highly resistant if the MIC was above 1 mg/l. For amoxicillin, cefotaxime and ceftriaxone, bacteria were considered to be sensitive if the MIC was 0.5 mg/l or less, of intermediate resistance if the MIC was above 0.5 mg/l but no greater than 2 mg/l, and highly resistant if the MIC was greater than 2 mg/l.

Serum PCT levels were determined using an immunoluminometric assay (Brahms Diagnostica, Berlin, Germany) with a limit of detection of 0.07 mg/ml.

### Treatment and course of illness

The efficacy of initial antibiotic treatment was assessed on the basis of *in vitro *bacterial sensitivity to antibiotics, bacteriological analysis of CSF samples drawn 48–72 hours after treatment initiation, and clinical course. The nature and duration of antibiotic treatment, and any modifications to this, were recorded. Mortality and sequelae were assessed at 30 days.

### Statistical analysis

Results are expressed as mean ± standard deviation or as median (interquartile range). The box plots are presented with the interquartile range. The values at day 0 (D0; admission) and day 2 (D2) were compared using Wilcoxon's nonparametric test for repeated measurements for quantitative parameters and the χ^2 ^test for qualitative parameters, with the threshold of significance set at *P *< 0.05.

## Results

During the study period (82 months), 59 patients presenting with ABM were admitted to the emergency department. Eleven patients were excluded for the following reasons: antibiotic treatment before admission (*n *= 6), presence of another site of infection (pneumopathy, *n *= 2; spontaneous bacterial peritonitis, *n *= 1), and serum PCT concentration 0.5 mg/ml or less on admission (one ABM due to pneumococci, one ABM due to unidentified bacteria).

The clinical characteristics of the 48 patients are summarized in Table 1. The mean interval between admission and lumbar puncture was 90 ± 40 min and the mean time elapsing from admission to injection of the first dose of antibiotic was 120 ± 70 min.

The microbiological results obtained are shown in Table 2. Among the 21 pneumococcal infections documented, 13 isolates (62%) were sensitive to penicillin, six (29%) were of intermediate resistance and two (10%) were resistant. Among the six strains with intermediate resistance to penicillin, four were sensitive to amoxicillin or to ceftriaxone, and two exhibited intermediate resistance. The two strains resistant to penicillin exhibited intermediate resistance to amoxicillin or to ceftriaxone. The initial antibiotic treatment comprised amoxicillin (150–200 mg/kg per day), ceftriaxone (60–80 mg/kg per day), or a combination of these. All of the pneumococcal strains with reduced sensitivity to penicillin were at least exposed to ceftriaxone during the initial treatment, with analysis of CSF samples drawn between 48 and 72 hours after the start of treatment showing sterilization in all cases. Antibiotic treatment was simplified eight times out of 21 on the basis of the results of microbiological analysis of CSF samples.

All of the other bacteria identified were sensitive to amoxicillin, with the initial antibiotic therapy being appropriate in all cases. The treatment was simplified between 24 and 72 hours after the start of treatment in six patients out of 24 on the basis of the results of microbiological analysis of the CSF.

For the three patients with a CSF culture not showing any evidence of bacteria, antibiotic treatment with amoxicillin and ceftriaxone was started on admission and continued for 15–20 days.

**Figure 1:**
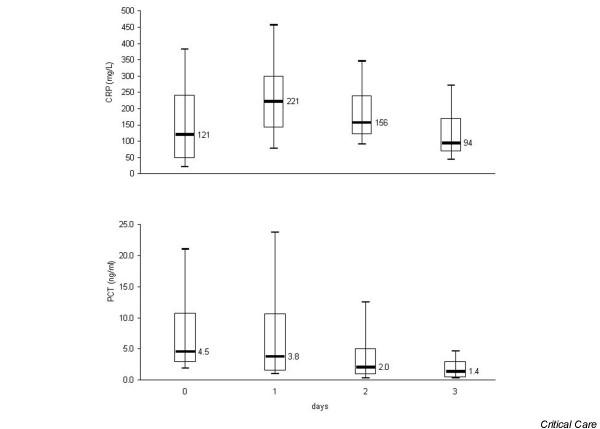
Evolution of CRP and PCT levels over 72 hours. Change in serum levels of C-reactive protein (CRP) and procalcitonin (PCT) from admission (day 0) to 72 hours after the start of treatment.

The changes in serum and CSF cytochemical parameters are shown in Tables 3 and 4 and in Fig. 1. With regard to the CSF, only the lactate concentration differed significantly between D0 and D2. Sterilization of the CSF was noted in the 34 patients who underwent a second lumbar puncture. In the 14 patients who did not undergo a repeat lumbar puncture, the duration of antibiotic treatment was 12–16 days, resulting in cure in all cases, and the duration of hospital stay was between 13 and 18 days. With respect to serum parameters, the decrease in PCT level was the only significant difference observed between D0 and D2.

Among the 48 patients, five patients (9%) died between 12 and 28 days after their admission to hospital. Only one of these patients was younger than 75 years. All of these patients underwent a second lumbar puncture during treatment, with analysis of the resulting sample showing sterilization of the CSF in all cases, which was confirmed by a third lumbar puncture in three of the five patients. A serum PCT concentration below 0.5 ng/ml was observed in all patients between 6 and 9 days after admission. The cause of death was multiple organ failure (*n *= 1), cerebral thrombophlebitis (*n *= 1) and cerebral oedema (*n *= 3). Four patients had neurological sequelae at 30 days.

## Discussion

In the present study a significant and early decrease in serum PCT concentration was associated with cure of meningitis. In contrast, analysis of CSF showed a significant decrease only in lactate concentration between 48 and 72 hours after the first lumbar puncture.

The value of repeat lumbar puncture at 48 hours remains debatable, and second-line antibiotic treatment is based essentially on the MIC of various antibiotics for the bacteria identified or on the clinical course [2,6,12,13,18]. Apart from the microbiological data, the CSF parameters traditionally described during ABM appear to be little modified by appropriate antibiotic therapy within 48 hours. Blazer and coworkers [16], studied the effect of antibiotic treatment on the CSF parameters of 68 children presenting with ABM. None of the cytochemical parameters studied (proteins, glucose, total and polymorphonuclear leucocytes) exhibited a significant decrease between the first lumbar puncture and a second lumbar puncture performed 44–68 hours after the start of antibiotic therapy, whereas two bacteria were still detectable in the repeat CSF samples drawn. Similar findings were reported by Bland and coworkers [19] concerning the changes in these cytochemical parameters after 24–72 hours of treatment for ABM in 15 children.

Different results were obtained in an animal study [20]. In five sheep, treatment for an experimentally induced meningitis due to *Escherichia coli *resulted in a rapid decrease in polymorphonuclear leucocyte count in the CSF, which was associated with an increase in glucose concentration and a decrease in protein concentration. However, in that study antibiotic treatment was administered intrathecally. In the present study there was no significant decrease in the polymorphonuclear leucocyte count or protein concentration in the CSF after 48–72 hours of appropriate antibiotic treatment. The glucose concentration measured in the CSF remained stable, but there was a significant decrease in lactate concentration.

Although numerous articles have demonstrated the value of assaying lactate during the course of ABM [19,21-27], few data exist concerning the changes in this parameter during the treatment of this disease. In 21 patients with ABM, Gontroni and coworkers [23] showed a rapid decrease in lactate concentration in the CSF during the first 24 hours of treatment. Gould [22], Bland [19] and Genton [21] and their groups obtained similar results concerning the change in lactate concentration in CSF after 24–72 hours of treatment in 6, 15 and 25 patients with ABM, respectively. In the study reported by Bland and coworkers [19], the mean lactate concentration in the CSF was 75.1 ± 6.6 mg/100 ml at the time of the first lumbar puncture and 49.5 ± 5.7 mg/100 ml after 24–72 hours of treatment.

With regard to the changes in serum parameters, the present study revealed a rapid decrease in PCT concentration within the first 24 hours of treatment, which was accompanied by an increase in CRP, with the level of CRP diminishing only after 2–3 days.

In 1993, Assicot and coworkers [17] demonstrated that serum PCT concentration was a marker of infectious states of bacterial origin in children, exhibiting a rapid decrease following antibiotic treatment. Although several studies have demonstrated the value of serum PCT concentration in the differential diagnosis of ABM and viral or aseptic meningitis [28-31], few data are available concerning the change in serum PCT during treatment for ABM. Schwartz and colleagues [31] reported a reduction in median serum PCT concentration from 1.75 mg/ml at baseline to 1.05 mg/ml after 48 hours of treatment in 11 patients with ABM. In three of these patients, the PCT concentration remained unchanged, or increased, in conjunction with an unfavourable clinical course. In the study reported by Gendrel and coworkers [28], conducted in eight children receiving treatment for ABM, the serum PCT concentration diminished within 24 hours of treatment in all but two cases.

Although appropriate antibiotic therapy appears to be correlated with a rapid decrease in PCT levels, the absence of patients receiving an inappropriate treatment in our series did not allow us to determine the change in PCT levels under these circumstances. What are the arguments in support of a relationship between decrease in PCT levels and appropriate antibiotic treatment? Smith and coworkers [32] investigated the value of PCT in 43 patients presenting with melioidiosis of various grades of severity. Among the 16 patients with a severe infection, 13 exhibited a decrease in PCT levels from the first day of treatment. In the three other patients an increase in PCT levels was observed in relation to infectious complications (pulmonary abscess, septic arthritis, splenic abscess). In two patients the *Pseudomonas pseudomallei *infection detected was resistant to the initial antibiotic therapy.

Although the change in serum levels of CRP has been shown to be of value for tracking the course of a bacterial infection during treatment [33,34], the characteristics of this protein are such that its concentration reaches a maximum only after 24–48 hours [35]; this is in contrast to PCT, which attains a peak serum concentration more rapidly. After injection of endotoxin, the peak serum concentration of PCT is reached within approximately 8 hours [36].

Certain limitations of the present study should be mentioned. This was a descriptive study of the variation in serum PCT concentrations over time in patients who had received appropriate antibiotic treatment from the moment they were admitted to hospital. We currently have no data on changes in serum PCT levels occurring in patients who did not receive suitable treatment. Two patients with bacterial meningitis were not included in the study on the grounds that they presented with a serum procalcitonin level below 0.5 ng/ml on admission. At present there is no clear explanation for this finding. Several studies have reported low levels of serum PCT during ABM [30,31,37]. For the most part, this occurred in patients presenting with bacterial meningitis caused by intracellular bacteria or nosocomial infections [31,37].

## Conclusion

The change in serum PCT level during treatment for community-acquired ABM appears to be a valuable parameter for evaluating the efficacy of antibiotic therapy. This hypothesis needs confirmation, particularly in patients presenting with bacterial meningitis that is not microbiologically documented.

## Key messages

• 	After appropriate antibiotic treatment, serum PCT level decrease within the first 24 hours.

• 	After appropriate antibiotic teatment, serum CRP level decrease between days 2 and 3.

• 	The value of repeat lumbar puncture at 48 hours remains debatable.

• 	We have no data on changes in serum PCT levels in patients who do not receive an appropriate antibiotic.

• 	Some patients presenting with ABM have a low serum PCT level.

## Abbreviations

ABM = acute bacterial meningitis; CRP = C-reactive protein; CSF = cerebrospinal fluid; MIC = minimum inhibitory concentration; PCT = procalcitonin.

## Competing interests

The author(s) declare that they have no competing interests.

## Authors' contributions

AV conceived of the study, and participated in its design and coordination and drafted the manuscript. PG participated in the inclusion and treatment of patients and drafted the manuscript. SG performed the statistical analysis. BT participated in the inclusion and treatment of patients. FR participated in the inclusion and treatment of patients. OM participated in the inclusion and treatment of patients. AC carried out the the microbiology. CL carried out the immunoassays. FZ participated in the design of the study and drafted the manuscript. JCB helped to draft the manuscript. All authors read and approved the final manuscript.

## Figures and Tables

**Table 1:** Patient characteristics on admission

Characteristics	Number of patients (*n *= 48)
Demographic characteristics	
Age (years; mean ± SD)	55 ± 21
Patients >75 years (*n *[%])	8 (17)
Male (*n *[%])	21 (44)
Duration of symptoms (hours; *n *[%])	
<12 hours	4 (8)
12–23 hours	15 (31)
24–48 hours	10 (21)
>48 hours	19 (40)
Clinial characteristics (*n *[%])	
Fever (>38°)	43 (90)
Headache	29 (60)
Nuchal rigidity	35 (73)
Seizures	4 (8)
Purpura	5 (10)
Focal neurological deficit	5 (10)
Glasgow Coma Scale score (mean ± SD)	13 ± 2
Simplified Acute Physiology Score II (mean ± SD)	18 ± 10

SD, standard deviation.

**Table 2:** Bacteriology of CSF on admission

Organism	CSF Gram stain (*n *= 48)	Culture (*n *= 48)
*Streptococcus pneumoniae *(*n *[%])	12 (25)	21 (44)
*Neisseria meningitidis *(*n *[%])	6 (12)	9 (19)
Other streptococci (*n *[%])	0	5 (10)
*Listeria monocytogenes *(*n *[%])	0	6 (13)
*Haemophilus influenzae *(*n *[%])	0	2 (4)
*Escherichia coli *(*n *[%])	0	1 (2)
*Staphylococcus aureus *(*n *[%])	0	1 (2)
Total (*n *[%])	18 (38)	45 (94)

CSF, cerebrospinal fluid.

**Table 3:** Cytochemical parameters of CSF and CSF/serum ratio on admission and after 2–3 days of treatment

Parameter	Day 0 (admission; *n *= 48)	Day 2 (2–3 days of treatment; *n *= 34)
Leucocyte count (cells/mm^3^)	757 (366–2730)	580 (309–2025)
Polymorphonuclear leucocyte count (cells/mm^3^)	605 (258–2482)	417 (254–1762)
Protein level (g/l)	4.2 (2–6.2)	3.9 (1.8–5)
Glucose CSF level (mmol/l)	2.4 (0.8–3.6)	2.5 (1.2–3)
CSF/serum glucose ratio	0.31 (0.1–0.48)	0.35 (0.17–0.5)
Lactate CSF level (mmol/l)	8.74 (5.5–13)	5* (3–9)
CSF/serum lactate ratio	3.22 (2.4–4.7)	2.73 (1.5–3.3)

Values are expressed as median (interquartile range). CSF, cerebrospinal fluid. *P < 0.001.

**Table 4:** Change in serum biological parameters from admission to day 4 of treatment

Parameter	Day 0	Day 1	Day 2	Day 3	Day 4
Leucocyte (10^9^/l)	14.2 (10–19)	14 (11.8–18)	13.5 (11–17)	10.5 (8.5–12)	10.2 (8–13)
Fibrinogen (g/l)	4.8 (4–6.4)	6.2 (5–7.5)	6.2 (5.6–8.2)	6.4 (5.8–8.4)	6.3 (5–8)
C-reactive protein (mg/l)	120 (48–241)	221 (141–299)	156 (121–240)	93 (67–170)	82 (43–130)
Procalcitonin ng/ml)	4.5 (2.8–10.8)	3.8 (1.5–10.7)	2* (0.9–5)	1.4 (0.4–3)	0.7 (0.4–1.3)

Values are expressed as median (interquartile range). *P < 0.0001 versus day 0.
